# Inhibition of neuroinflammatory nitric oxide signaling suppresses glycation and prevents neuronal dysfunction in mouse prion disease

**DOI:** 10.1073/pnas.2009579118

**Published:** 2021-03-02

**Authors:** Julie-Myrtille Bourgognon, Jereme G. Spiers, Sue W. Robinson, Hannah Scheiblich, Paul Glynn, Catharine Ortori, Sophie J. Bradley, Andrew B. Tobin, Joern R. Steinert

**Affiliations:** ^a^Centre for Immunobiology, University of Glasgow, Glasgow, G12 8TA, United Kingdom;; ^b^Department of Biochemistry and Genetics, La Trobe Institute for Molecular Science, La Trobe University, VIC 3083, Melbourne, Australia;; ^c^Medical Research Council Toxicology Unit, University of Leicester, Leicester, LE1 9HN, United Kingdom;; ^d^Department of Neurodegenerative Disease and Geriatric Psychiatry/Neurology, University of Bonn, Bonn 53127, Germany;; ^e^School of Pharmacy, University of Nottingham, Nottingham NG7 2RD, United Kingdom;; ^f^Centre for Translational Pharmacology, Veterinary and Life Sciences, University of Glasgow, Glasgow, G12 8QQ, United Kingdom;; ^g^School of Life Sciences, Queen’s Medical Centre, University of Nottingham, Nottingham, NG7 2UH, United Kingdom

**Keywords:** neurodegeneration, nitric oxide, neuroinflammation, glycation, prion aggregation

## Abstract

Several neurodegenerative diseases associated with protein misfolding (Alzheimer’s and Parkinson’s disease) exhibit oxidative and nitrergic stress following initiation of neuroinflammatory pathways. Associated nitric oxide (NO)-mediated posttranslational modifications impact upon protein functions that can exacerbate pathology. Nonenzymatic and irreversible glycation signaling has been implicated as an underlying pathway that promotes protein misfolding, but the direct interactions between both pathways are poorly understood. Here we investigated the therapeutic potential of pharmacologically suppressing neuroinflammatory NO signaling during early disease progression of prion-infected mice. Mice were injected daily with an NO synthase (NOS) inhibitor at early disease stages, hippocampal gene and protein expression levels of oxidative and nitrergic stress markers were analyzed, and electrophysiological characterization of pyramidal CA1 neurons was performed. Increased neuroinflammatory signaling was observed in mice between 6 and 10 wk postinoculation (w.p.i.) with scrapie prion protein. Their hippocampi were characterized by enhanced nitrergic stress associated with a decline in neuronal function by 9 w.p.i. Daily in vivo administration of the NOS inhibitor L-NAME between 6 and 9 w.p.i. at 20 mg/kg prevented the functional degeneration of hippocampal neurons in prion-diseased mice. We further found that this intervention in diseased mice reduced 3-nitrotyrosination of triose-phosphate isomerase, an enzyme involved in the formation of disease-associated glycation. Furthermore, L-NAME application led to a reduced expression of the receptor for advanced glycation end-products and the diminished accumulation of hippocampal prion misfolding. Our data suggest that suppressing neuroinflammatory NO signaling slows functional neurodegeneration and reduces nitrergic and glycation-associated cellular stress.

## Significance

We present evidence that alleviating nitrergic stress during early phases of neurodegeneration reduces neuroinflammatory posttranslational nitric oxide signaling and glycation-assisted dysfunction in the hippocampus of prion-diseased mice, a mechanism which might be applicable to other protein-misfolding neurodegenerative conditions. We confirmed that pharmacological suppression of nitrergic activity reduces the formation of advanced glycation end-products, diminishes prion protein misfolding, and averts neuronal dysfunction. This intervention could present an approach to diminish the detrimental neuroinflammatory effects seen in neurodegeneration and highlights nitrergic stress and glycation signaling as putative targets for disease-modifying treatments. The correlation between protein glycation and prion misfolding—as reported for several other misfolding proteins—links NO signaling to the neuroprotective effects seen following NOS inhibition.

Diseases associated with old age, including Alzheimer’s disease (AD) and other forms of dementia, are increasing in prevalence, and although symptomatic treatments exist, no cure to prevent the disease progression has yet been described. Neurodegenerative diseases associated with the accumulation of misfolding proteins including AD, Parkinson’s disease (PD), and various forms of prion disease share many common features such as up-regulation of neuroinflammatory signaling (1). In particular, such signaling can compromise synaptic plasticity (2) in early degeneration representing an initiation phase for dysfunction. It is well established that cellular stress signaling in neurodegenerative diseases is associated with aberrantly elevated levels of nitric oxide (NO) and activation of oxidative/nitrergic stress pathways (3–5). NO has both physiological and pathological roles in the nervous system where it is synthesized by NO synthases (neuronal NOS [nNOS], inducible NOS [iNOS], and endothelial NOS [eNOS]). Excessive NO production reported in prion disease, AD, and PD is inducing aberrant posttranslational protein modifications and uncontrolled neurotoxic nitrergic signaling (6–9). Protein cysteine residues can be directly and reversibly S-nitrosylated by NO (9) which can lead to protein dysfunction to facilitate disease progression (10, 11). NO also reacts with free radicals such as superoxide anion O_2_^•**−**^ resulting in the generation of peroxynitrite (ONOO^**−**^) which accumulatively induces protein nitrotyrosination, the irreversible chemical addition of a nitro group to protein tyrosine residues generating 3-nitrotyrosine (3-NT), which can result in a loss of physiological protein function (12). Therefore, preventing disease-relevant, in particular, iNOS-mediated NO generation presents a strong and promising therapeutic target across many neurological diseases (13–15).

In neurodegenerative diseases, oxidative and nitrergic stress is believed to be an initiator of many pathological pathways further augmenting underlying neurodegeneration (16–18). For example, 3-NT of β-amyloid (Aβ) oligomers presents a direct pathway for increasing oligomer stability and toxicity (12, 19). Interestingly, Aβ_42_ oligomerization also plays a key role by promoting nitrergic stress, i.e., inducing 3-NT of numerous neuronal proteins (20). One targeted protein is the enzyme triose-phosphate isomerase (TPI) which regulates the glycolytic flow by interconverting dihydroxyacetone phosphate (DHAP) and glyceraldehyde 3-phosphate (GAP). The nitrergic posttranslational modification of TPI induced by the Aβ_42_ peptide reduces its enzymatic activity (21) leading to a shift of balance of the metabolites toward DHAP formation. Subsequently, DHAP transforms spontaneously into the cytotoxic metabolic by-product methylglyoxal (22, 23). A nitrooxidative environment favors high levels of 3-nitrotyrosinated TPI in AD patients (21), and as a result, inhibition of this enzyme leads to nonenzymatic methylglyoxal-mediated generation of protein glycation (21) and formation of advanced glycation end-products (AGE).

The production of AGE is irreversible and resulting glycated proteins have a propensity to aggregate (24) while exhibiting resistance to protease degradation. Notably, protein glycation is a marker of aging, and it promotes inflammation via the overproduction of intracellular reactive oxygen species (ROS) in addition to impairing proteasomal activities. The resulting AGE-mediated induction of oxidative stress thereby leads to augmented oxidative damage in the brain presenting a positive feedback loop of neurotoxic pathways in different protein misfolding diseases (25, 26). In AD, the aggregation and deposition of AGEs have been observed in tau positive intracellular aggregates and Aβ plaques (27, 28) with the polymerization of Aβ being significantly accelerated by AGE-mediated protein cross-linking (29). Glycation at specific residues causes a profound impact on the ability to form amyloid fibrils (30, 31) confirming a contributing role of this modification in aggregation. AGEs are principally recognized by the receptor for AGE (RAGE) that belongs to the immunoglobulin gene superfamily. RAGE is found on the surface of numerous cells types including microglia, monocytes, astrocytes, neurons, and endothelial cells (32). RAGE activation results in the stimulation of nicotinamide adenine dinucleotide phosphate (NADPH) oxidase (33), an enzyme that produces superoxide radicals. It also induces the production of cytokines via NF-κB expression, followed by up-regulation of inflammatory pathways (32). Importantly, AGE-formation and RAGE expression are increased in brains from Creutzfeldt Jakob disease (CJD) patients and are associated with enhanced oxidative and nitrergic stress (34, 35) suggesting the possibility that AGE-mediated modifications might, in part, play a role in the accumulation of the aberrant prion protein.

In this study, we investigated neurodegeneration induced by prion misfolding to assess the role of nitrergic stress caused by excess neuroinflammatory production of NO. We established that the decline of hippocampal CA1 pyramidal neuronal function is associated with increased nitrergic stress and reduced levels of antioxidants and that prolonged in vivo treatment with an NOS inhibitor reduced prion protein aggregation and diminished aspects of neurodegenerative signaling such as neuronal and synaptic dysfunction. We further found that blocking NO signaling prevented nitrotyrosination of TPI and RAGE up-regulation.

## Materials and Methods

### Mice and Prion Inoculation.

Hemizygous Tg37 mice that overexpress the cellular mouse prion protein (PrP^C^) have been used as described previously (36). Mice were fed ad libitum with a standard mouse chow. Tg37 mice aged 3 wk were inoculated by intracerebral injection into the right parietal lobe with 1% brain homogenate of Rocky Mountain Laboratory (RML) misfolded prion protein (PrP^Sc^) (36). Control mice received 1% normal brain homogenate (NBH), and hippocampi and cortices of both groups were used for analyses.

### Animal Drug Treatment.

RML-inoculated mice were treated (i.p.) with vehicle (5% glucose [control]) or N(ω)-nitro-l-arginine methyl ester (L-NAME, 20 mg/kg) daily from 6 wk postinoculation (w.p.i.) for 3 wk. This dosage provides an ∼70% inhibition of lipopolysaccharide-induced NO_x_ generation (via iNOS induction) in mice with an ED_50_ for L-NAME of 5 mg/kg (37). The presence of the L-NAME metabolite and active inhibitor of NOS in the brain, L-NOARG (38), was confirmed by high-performance liquid chromatography (HPLC; *SI Appendix*) at a concentration of 11.8 ± 1.1 nM/g tissue (*n* = 8 mice).

### Western Blotting.

Mouse hippocampi were dissected into ice-cold phosphate-buffered saline (PBS) containing protease inhibitors and phosphatase inhibitors (Complete, Roche Diagnostics), and samples were flash-frozen in liquid nitrogen until further analysis. All the following procedures were then performed at 4 °C. Frozen tissue was homogenized in ice-cold RIPA buffer (20 mM Tris [pH 7.4], 150 mM NaCl, 3 mM EDTA, and 1% Nonidet P-40) and 1× protease inhibitor mixture with a tissue homogenizer (IKA T10 *basic ULTRA*-*TURRAX*). Total protein concentration was measured by the Bradford method (BioRad Protein assay kit). Primary antibody details are shown in *SI Appendix*.

### Immunocytochemistry.

Mice were anesthetized with 3% isofluorane (2 L/min O_2_) and transcardially perfused with 20 mL of ice-cold PBS, followed by 20 mL of ice-cold 4% PFA. Following fixation, brains were immediately removed and further fixed overnight in 4% PFA at 4 °C. Brains were processed in paraffin wax and sliced at 5 µM using a microtome. Following antigen retrieval, sections were washed in TBS + 0.1% triton X-100 and blocked for 2 h at RT in TBS, 0.1% triton X-100, 10% goat serum, and 5% BSA. Following three washes, slices were mounted in Vectorshield hardset mounting medium with/without DAPI (Santa Cruz). Antibody and cell count analysis details are shown in *SI Appendix*. All images were taken using a Zeiss LSM 510 META NLO microscope with ZEN 2009 software (Zeiss).

### NADPH-Diaphorase Assay.

Mice were anesthetized and transcardially perfused with PBS then 4% PFA. The brain was removed and fixed for 24 h in PFA before being embedded in paraffin. Five-μm sections were cut and deparaffinized before being incubated in a mixture of 2.5 mg nitroblue tetrazolium, 10 mg β-NADPH, and 0.2% Triton X-100 in 10 mL of 0.05 M Tris buffer for 30 min at 37 °C. The reaction was terminated with 0.05 M Tris buffer washes.

### qRT-PCR.

Total RNA was extracted from isolated hippocampal tissue using RNeasy mini kit (Qiagen) and treated with deoxyribonuclease 1. For each tissue, 1 µg of total RNA was reverse transcribed using an iScript cDNA synthesis kit according to the manufacturer’s instructions (BioRad). Real-time PCR reactions were performed using 25 to 100 ng of template cDNA per reaction and Taqman gene expression “assay on demand” assays. The target primer/probes are presented in *SI Appendix*. Relative mRNA expression in each region was determined by normalizing to the average amount of hippocampus or cortex from NBH mice using the ΔΔCT method.

### Electrophysiology.

Brain slices were prepared from animals killed by cervical dislocation. Whole-cell patch recordings were made from visually identified CA1 neurons in acute brain slices (300 μm thick) of the hippocampus as described previously (39, 40). Data were recorded using a Multiclamp 700B amplifier (Molecular Devices). Stimulation, data acquisition, and analysis were performed using pClamp 10.4 and Clampfit 10.4 (Molecular Devices). An artificial cerebrospinal fluid (aCSF) was used for slice incubation and perfusion during recordings. A low-sodium aCSF was used during preparation of slices. Synaptic stimulation at the Schaffer collateral was achieved using an isolated stimulator via a bipolar platinum electrode. Solutions and protocols are detailed in *SI Appendix*.

### Ultrahigh-Performance Liquid Chromatography-Tandem Mass Spectroscopy.

Mass spectrometry studies were performed by Metabolon as described previously (8). Samples were prepared using the automated MicroLab STAR system (Hamilton Company). To remove protein, dissociate small molecules bound to protein or trapped in the precipitated protein matrix, and recover chemically diverse metabolites, proteins were precipitated with methanol under vigorous shaking for 2 min (Glen Mills GenoGrinder 2000) followed by centrifugation; see *SI Appendix* for further details.

### Statistical Analysis.

Results are expressed as the means ± SEM. Statistical comparisons were made by one-way analysis of variance (ANOVA) with Newman–Keuls posteriori analysis, two-way ANOVA with Bonferroni's multiple comparisons test, Kolmogorov–Smirnov test for nonparametric data distributions, or unpaired Student’s *t* test, as appropriate. All datasets passed the normality testing using the Shapiro–Wilk test. A *P* value smaller than 0.05 was considered as significant. All statistical tests were performed using GraphPad Prism v9 software.

### Study Approval.

All animal work conformed to the United Kingdom Home Office regulations. All procedures were conducted under a Home Office project license awarded to J.R.S. under the Animals (Scientific Procedures) Act 1986.

## Results

### Prion-Diseased Mice Show Signs of Neuroinflammation prior to Neuronal Dysfunction.

**Fig. 1.**
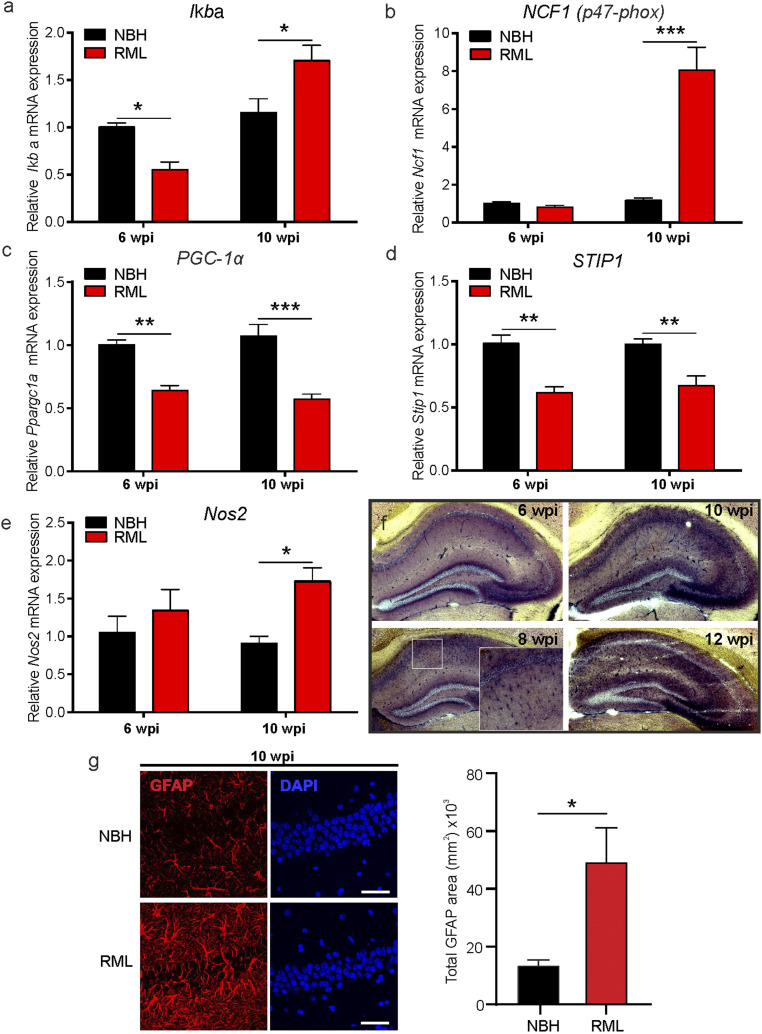
Neuroinflammation is enhanced early in prion disease. mRNA levels were determined at 6 and 10 w.p.i. in control (NBH) and prion-infected (RML) mice for the following genes: (*A*) *Iκbα*, (*B*) *NCF1*, (*C*) *PGC-1α*, (*D*) *STIP1*, and (*E*) *Nos2*. (*F*) NADPH diaphorase staining of prion-inoculated brains (RML) at 6 to 12 w.p.i. shows enhanced signals in disease detectable after 6 w.p.i. with strong signals in the CA1 and at later time points spreading across the whole hippocampus. (*G*) (*Left*) Immunocytochemistry images show representative GFAP and DAPI staining in the hippocampus of NBH and RML mice at 10 w.p.i. and (*Right*) GFAP signals presented as absolute area. (Scale bar: 50 μm.) Data are presented as mean ± SEM, *n* = 4 NBH and *n* = 7 RML mice, two-way ANOVA (*A*–*E*) and unpaired Student’s *t* test (*G*), *P < 0.05, **P < 0.01, ***P < 0.001.

Previously, we have found that in the hippocampus and cortex of prion-infected mice the neuronal metabolism, not only related to the NO signaling cascade, is aberrantly regulated at 10 w.p.i. (8). At this advanced time point we found evidence for numerous pathological markers such as high levels of misfolded prion protein (PrP^Sc^) in the hippocampus and cortex, enhanced glial fibrillary acidic protein (GFAP) expression associated with nitrergic and oxidative stress, and hippocampal neuronal loss as well as learning and memory deficits (40). However, as the disease is highly progressed by 10 w.p.i., we investigated in this study disease-relevant pathways which may present a potential target for disease rescue at earlier time points. We examined the contributions of neuroinflammatory and oxidative stress-related signaling in prion-infected mice (RML) at a time point prior to onset of reported disease symptoms (at 6 w.p.i.), as well as advanced disease stages (at 9 to 10 w.p.i.). We first tested gene expression for the inflammatory suppressor of NF-κB signaling, *Iκbα*, and found it to be down-regulated at 6 w.p.i. compared to NBH mice (*P* = 0.038, two-way ANOVA, *n* = 4 mice each; Fig. 1*A*). RNA expression for the peroxisome proliferator-activated receptor-gamma coactivator, PGC-1α, which controls the expression of genes related to the generation of ROS and prevents oxidative stress by reducing the production of ROS (41), is also down-regulated at this early stage of disease (*P* = 0.0015, two-way ANOVA, *n* = 4 mice each; Fig. 1*C*), as is the prion-interacting protein stress-inducible phosphoprotein 1, STIP1 (42) (*P* = 0.0013, two-way ANOVA, *n* = 4 mice each; Fig. 1*D*). At 10 w.p.i. we found a strong increase in mRNA levels for *Iκbα* (*P* = 0.012) as well as *NCF1* (*P* < 0.0001), one of the components of NADPH oxidase (p47-phox [Fig. 1 *A* and *B*], *n* = 4 mice each). Furthermore, *PGC-1α* (*P* < 0.0001, two-way ANOVA) and *STIP1* (*P* = 0.0047, two-way ANOVA) mRNA levels remain reduced at this later disease stage (Fig. 1 *C* and *D*). Importantly, *Nos2* mRNA (iNOS) is increased at 10 w.p.i. (*P* = 0.0266, two-way ANOVA; Fig. 1*E*) with *Nos1* (nNOS) and *Nos3* (eNOS) mRNA levels being strongly reduced at 6 and 10 w.p.i. (*SI Appendix*, Fig. S1). The enhanced iNOS activity is likely a result of activated astroglia signaling which we further illustrated using the NADPH diaphorase assay at 6 to 12 w.p.i. (Fig. 1*F*). We have previously shown that NBH mice lack any significant NADPH diaphorase signals at 10 w.p.i. (8). These data illustrate a strong increase of NOS (NADPH activity) from 8 w.p.i. onward in RML mice with signals reminiscent of activated astroglia in the CA1 region spreading to the whole hippocampus at 10 and 12 w.p.i. Further characteristics for up-regulated astroglia were detected by immunostaining for GFAP which showed a significantly stronger expression at 10 w.p.i. in RML hippocampi (*P* = 0.045, Student’s *t* test; Fig. 1*G*). Altogether, our data indicate that an early onset neuroinflammatory contribution to prion disease is detectable from 8 wk onward. Thus, our approach to start targeting NO signaling will focus on time points following 6 w.p.i. onward.

### Reducing Nitrergic Stress Prevents Neuronal and Synaptic Dysfunction in Neurodegeneration.

**Fig. 2.**
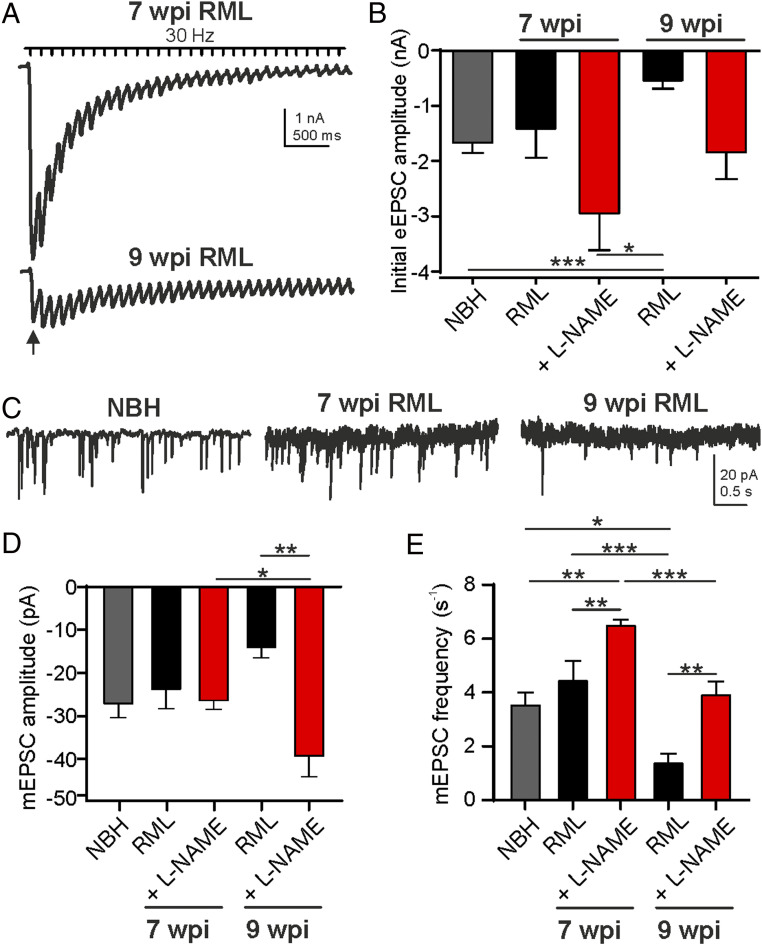
Decline in synaptic strength is prevented by NOS antagonism. (*A*) Representative eEPSC trains recorded at 30 Hz for 10 s in the CA1 pyramidal neurons from a RML mouse at 7 w.p.i. and RML mouse at 9 w.p.i. (*B*) Initial eEPSC amplitudes for NBH (gray) and vehicle-treated (black) and L-NAME–treated RML (red) mice at both time points. (*C*) Raw traces showing spontaneous release events for NBH and RML mice at indicated time points. mEPSC (*D*) amplitudes and (*E*) frequencies show disease-dependent decline (black) in RML mice. L-NAME treatment prevented the reduction in mEPSC amplitudes and frequencies (red). Data are presented as mean ± SEM, *n* = 4 NBH, *n* = 9 RML and *n* = 6 RML + L-NAME–treated mice with *n* = 4 to 10 neurons per mouse, one-way ANOVA, *P < 0.05, **P < 0.01, ***P < 0.0001.

We next asked whether interference with neuroinflammatory NO signaling prior to detectable prion misfolding might alleviate some of the neuronal symptoms reported in the disease. In order to address this question, subsequent studies specifically focused on RML mice and the effects of treatment since we did not find any indications for aberrant nitrergic signaling in NBH controls (Fig. 1). We quantified the electrophysiological phenotypes of CA1 pyramidal neurons and their Schaffer collateral-mediated synaptic inputs as a read-out of neuronal health in RML prion-infected mice treated with the NOS inhibitor, L-NAME, daily from 6 w.p.i. in comparison to age-matched vehicle-treated RML (all 7 and 9 w.p.i.) and NBH controls (7 w.p.i.). These time points were chosen to study the effects of NOS manipulation based on data presented in Fig. 1 at which we expect pathological neuroinflammation to become dominant. Fig. 2*A* shows representative Schaffer collateral-evoked excitatory postsynaptic currents (eEPSC) in pyramidal CA1 neurons in RML mice at 7 and 9 w.p.i. following stimulation by a 30-Hz train. The recordings illustrate a vast deterioration in neuronal function by 9 w.p.i. Mean initial eEPSC amplitudes (arrow) from NBH, vehicle- (RML), or L-NAME–treated RML mice are summarized in Fig. 2*B* showing a strong reduction at 9 wk. Following 3 wk of daily treatment with L-NAME, the decline in mean initial current amplitudes was prevented (NBH vs. RML 9 w.p.i., *P* < 0.0001, one-way ANOVA; Fig. 2*B*). Cumulative postsynaptic current analysis, a measure to estimate available vesicle pool sizes based on the cumulative eEPSC amplitude plots, revealed a larger vesicular pool size at 9 w.p.i. following L-NAME treatment (*P* = 0.043, two-way ANOVA; *SI Appendix*, Fig. S2) suggesting a reduced deterioration of synapse function following treatment. An important regulatory mechanism for synaptic vesicular release is the control of release probabilities. As a measure to determine the initial synaptic release probabilities, we assessed paired-pulse ratios (PPR, eEPSC_2_/eEPSC_1_ amplitude) at 33-ms interspike intervals of Schaffer collateral-stimulated eEPSCs in treated and untreated RML mice. PPR values of synaptic responses in hippocampi from untreated mice revealed a significant increase at 9 w.p.i. indicating a reduction in release probability over time (*P* = 0.0145, two-way ANOVA; *SI Appendix*, Fig. S2). L-NAME treatment, however, suppressed this increase in PPRs, which suggests that NOS inhibition prevents different aspects of synapse dysfunction. A further assessment criterion of neuronal health is the ability of synapses to spontaneously release vesicles. These spontaneous or miniature EPSCs (mEPSC) provide important signals for physiological neuronal homeostatic signaling (43). Consistent with above data, we found that mEPSC amplitudes were increased following 3 wk of L-NAME treatment compared to vehicle treatment (RML 9 w.p.i. vs. RML 9 w.p.i. + L-NAME, *P* = 0.0015) and compared to 1 wk of L-NAME treatment (RML 7 w.p.i. + L-NAME vs. RML 9 w.p.i. + L-NAME, *P* = 0.0004, one-way ANOVA; Fig. 2 *C* and *D*). Furthermore, spontaneous firing frequencies were reduced at 9 w.p.i. compared to NBH (*P* = 0.0146) and RML at 7 w.p.i. (*P* = 0.0007; Fig. 2 *C* and *D*), with L-NAME treatment increasing firing rates at both time points relative to vehicle treatment (7 w.p.i., *P* = 0.0068; 9 w.p.i., *P* = 0.0024, one-way ANOVA). Analyzing the relative cumulative distributions of mEPSC amplitudes and interspike intervals (ISI) of spontaneous release events revealed a strong leftward shift in amplitudes and rightward shift in ISI values in diseased mice at 9 w.p.i. compared to 7 w.p.i. (*SI Appendix*, Fig. S2; amplitudes, *P* < 0.0001, D = 0.571; ISI, *P* < 0.0001, D = 0.738; Kolmogorov–Smirnov test, 9 vs. 7 w.p.i.). A protective effect of L-NAME treatment was observed at 9 w.p.i. (*SI Appendix*, Fig. S2; amplitudes, *P* < 0.0001, D = 0.571; ISI, *P* < 0.0001, D = 0.683; Kolmogorov–Smirnov test, 9 w.p.i. vs. 9 w.p.i. + L-NAME treatment).

**Fig. 3.**
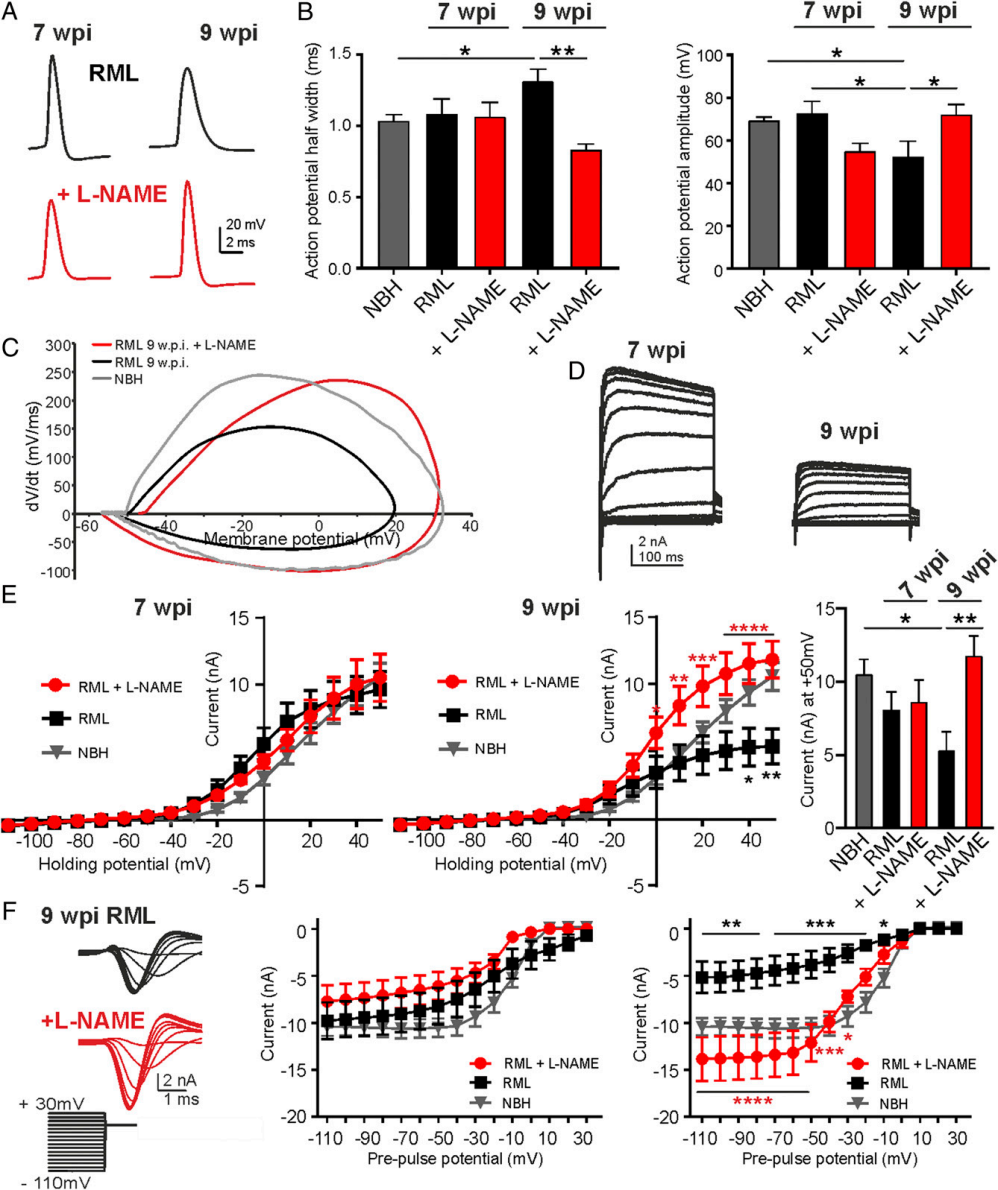
Neuronal function is protected by NOS antagonism. (*A*) Representative current-evoked AP recordings. (*B*) AP half-width and amplitudes were analyzed at 7 and 9 w.p.i. for NBH (gray), vehicle-treated (black), and L-NAME–treated RML (red) mice. (*C*) Phase plots for averaged APs from NBH, RML, and RML + L-NAME–treated mice. Note the strongly reduced upstroke values (dV/dt) and amplitudes in RML mice which were recovered following L-NAME treatment. (*D*) Representative recordings of voltage-gated potassium currents at 7 and 9 w.p.i. in RML mice. (*E*) IV relationships at 7 and 9 w.p.i. for NBH, RML, and RML + L-NAME–treated mice. Treatment prevented the decline in voltage-gated potassium currents at 9 w.p.i. (red). (*Right*) Mean currents at +50 mV. (*F*) Voltage-gated sodium currents decline at 9 w.p.i. in RML mice with L-NAME treatment (red) preventing the reduction in currents at 9 w.p.i. (representative recordings at 9 w.p.i.). Data are presented as mean ± SEM, *n* = 4 NBH, *n* = 9 RML and *n* = 6 RML + L-NAME–treated mice with *n* = 4 to 6 neurons per mouse, one-way ANOVA, *P < 0.05, **P < 0.01, ***P < 0.001, ****P < 0.0001.

We next assessed neuronal function by measuring the ability of CA1 pyramidal neurons to generate current-evoked action potentials (AP) and characterized the underlying whole-cell potassium and sodium currents. Both ion channel function and AP propagation play crucial roles in regulating neuronal performance and allow the neurons to sustain reliable information transmission. AP parameters such as half-width and amplitude are main characteristics, both predominately determined by potassium and sodium channel activities, respectively. L-NAME treatment averted the increase in AP half-width at 9 w.p.i. (NBH vs. RML 9 w.p.i., *P* = 0.0174; RML 9 w.p.i. vs. RML 9 w.p.i. + L-NAME, *P* = 0.0008, one-way ANOVA; Fig. 3 *A* and *B*) indicating a disease-linked reduction in potassium currents, prevented by NOS inhibition. AP amplitudes were reduced at 9 w.p.i. (NBH vs. RML 9 w.p.i., *P* = 0.0311; RML 7 w.p.i. vs. 9 w.p.i., *P* = 0.0262, one-way ANOVA) and increased at 9 w.p.i. following L-NAME treatment (Fig. 3 *A* and *B*; RML 9 w.p.i. vs. RML 9 w.p.i. + L-NAME, *P* = 0.0336, one-way ANOVA), suggesting an involvement of sodium channel activities which provide the predominant driving force for the depolarization during an AP (44). A change of AP waveforms becomes more apparent in the generated phase plots of APs from NBH, RML, and RML + L-NAME–treated mice. The phase plots show that the maximal upstroke (dV/dt) values of an AP, where the inflowing sodium current is highest, has increased drastically following L-NAME treatment compared to AP upstroke values in RML mice (Fig. 3*C*) which would be indicative of smaller sodium currents and directly translates into smaller AP amplitudes (44).

We next assessed both voltage-gated potassium and sodium currents directly by voltage clamp. Current–voltage (IV) relationships showed a strong decline of outward potassium currents (Fig. 3 *D* and *E*; NBH vs. RML 9 w.p.i., *P* < 0.05 at +40 mV and +50 mV, two-way ANOVA) and inward sodium currents at 9 w.p.i. in RML mice (Fig. 3*F*; NBH vs. RML 9 w.p.i., *P* < 0.05 between −110 and −10 mV, two-way ANOVA), in agreement with the observed changes in AP parameters, with L-NAME treatment preventing the decline of both currents at 9 w.p.i. (potassium current, RML 9 w.p.i. vs. RML 9 w.p.i. + L-NAME, *P* < 0.05 between 0 and +50 mV; sodium current, RML 9 w.p.i. vs. RML 9 w.p.i. + L-NAME, *P* < 0.05 between −110 and −30 mV, two-way ANOVA). The data on neuronal physiology were supported by measurements of neuronal proteins levels involved in synaptic function and neuronal fidelity. Protein levels of several synaptic markers, including MUNC 18, SNAP-25, and complexin 1/2, were reduced with others showing strong tendencies of lower expression (synaptophysin, synaptobrevin, and synapsin) at 9 w.p.i. (*SI Appendix*, Fig. S3). Protein expression of the voltage-gated potassium channel Kv3.1, one of the main conductances contributing to the presented IV relationships in Fig. 3*D* and determinants of AP half-width, declined at 9 w.p.i. Altogether, the data illustrate the broad neuronal dysfunction at late disease stages and the potent effects of L-NAME treatment to prevent several aspects of the dysfunction via suppression of excessive NO signaling in vivo.

### Protein 3-Nitrotyrosination Is Driven by Oxidative Stress and Enhances Protein Glycation in Prion Disease.

**Fig. 4.**
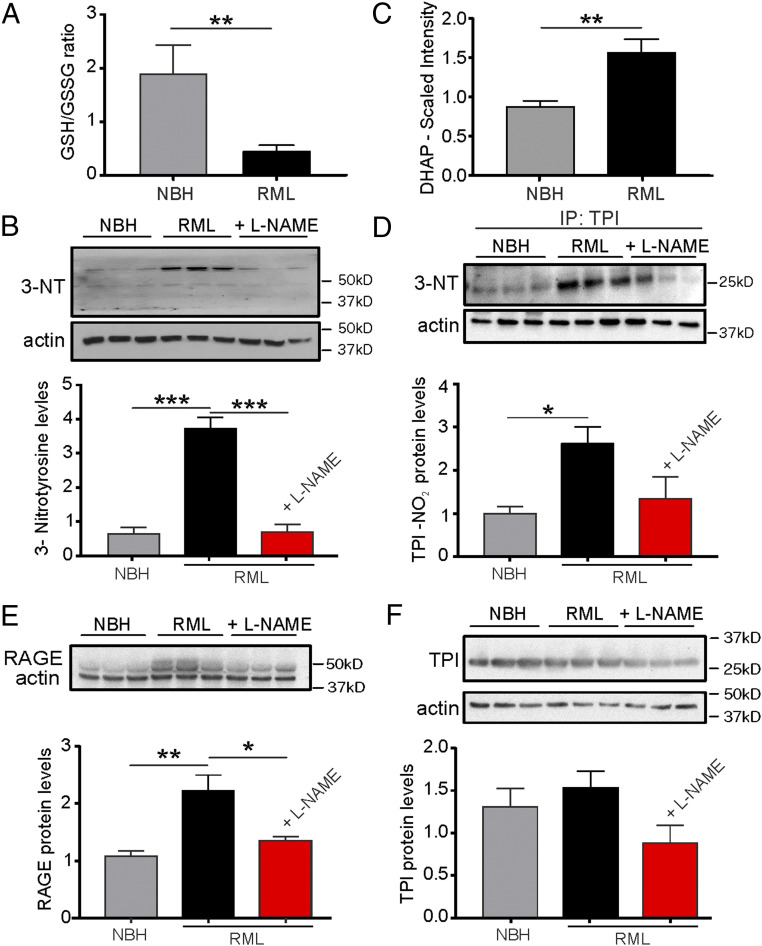
NOS inhibition reduces protein 3-nitrotyrosination and glycation signaling. (*A*) The ratio of reduced/oxidized glutathione is diminished in hippocampal tissue from RML mice at 10 w.p.i.. (*B*) Levels of 3-NT proteins are enhanced in RML and reduced following L-NAME treatment of RML mice. (*C*) DHAP levels are increased in the hippocampus of RML mice. (*D*) The 3-NT formation of triose-phosphate isomerase (TPI) is increased in RML mice and reduced to control NBH levels following L-NAME treatment of RML mice. (*E*) RAGE levels (upper band; lower band represents actin) are enhanced in RML hippocampi. (*F*) Total TPI levels are not affected by prion disease or treatment. Data are presented as mean ± SEM, *n* = 6 NBH, *n* = 9 RML mice (*A* and *C*) and *n* = 3 mice each in *B* and *D*–*F.* Unpaired Student’s *t* test, **P < 0.01 (*A* and *C*), one-way ANOVA, *P < 0.05, **P < 0.01, ***P < 0.001 (*B* and *D*–*F*)

Protein 3-nitrotyrosination is characteristic of protein-misfolding neurodegeneration (22, 45). The 3-NT occurs under conditions of oxidative stress and aberrant nitrergic activity. We confirmed a decreased ratio of reduced to oxidized glutathione (GSH/GSSG) in prion-diseased mice at 9 w.p.i. (*P* = 0.0039, control NBH vs. RML mice, Student’s *t* test; Fig. 4*A*) providing evidence for enhanced oxidative stress as one hallmark of the disease and confirming previously reported increases in oxidative stress levels in prion-diseased mice (8). Together with the enhanced levels of nitrergic stress associated with augmented expression levels of iNOS mRNA (Fig. 1), we found total amounts of 3-nitrotyrosinated proteins to be greater than fivefold elevated in prion-infected mice compared to NBH controls at 9 w.p.i. (*P* = 0.0005, control NBH vs. RML, one-way ANOVA; Fig. 4*B*). Importantly, we could not detect these elevated levels of 3-NT following treatment with L-NAME (*P* = 0.0005, RML + L-NAME vs. RML, one-way ANOVA; Fig. 4*B*).

Both AD and CJD are associated with neurotoxic glycation signaling as well as a strong presence of AGEs (22, 23, 35). The predominant neuronal precursor to induce protein glycation is DHAP which spontaneously forms methylglyoxal, a strong glycating agent (46). We have measured DHAP by mass spectrometry and found levels to be approximately twofold increased (*P* = 0.0027, Student’s *t* test; Fig. 4*C*) in the hippocampus of prion-infected mice. As TPI is the upstream enzyme in the cascade to interconvert DHAP into GAP, a reduction of its enzymatic activity would be responsible for the observed accumulation of DHAP. It has been previously reported that mutations of TPI carrying the tyrosine substitutions Y165F and Y209F, both of which are located at the catalytic center, render this enzyme inefficient when expressed in human neuroblastoma cells thereby shifting the balance between GAP and DHAP toward the latter and reducing cell viability (23). Based on these findings, we tested whether we could detect a nitrergic posttranslational modification of TPI in prion-diseased mice that would ultimately be responsible for an accumulation of DHAP. Indeed, when performing immunoblotting for 3-nitrotyrosination after having immunoprecipitated TPI, we detected an increase of 3-NT TPI protein levels in diseased mice (Fig. 4*D*; *P* = 0.033, one-way ANOVA, RML vs. NBH). Importantly, treatment with L-NAME prevented the increase in 3-NT TPI resulting in levels similar to NBH controls (Fig. 4*D*; *P* = 0.484, one-way ANOVA, NBH vs. RML + L-NAME). Total amounts of TPI protein were unchanged (Fig. 4*F*; *P* = 0.163, one-way ANOVA). The production of AGE following TPI nitrotyrosination and DHAP accumulation is directly linked to the activation of the receptor for AGE (RAGE), and the expression of RAGE reflects evidence for AGE signaling (47). To test whether we could corroborate enhanced AGE signaling in prion mice we examined RAGE expression and found that prion-diseased mice exhibited markedly enhanced levels of RAGE (Fig. 4*E*; *P* = 0.0074, one-way ANOVA). We tested the effects of NOS inhibition on RAGE expression and found that treatment with L-NAME ameliorated these increases (Fig. 4*E*; *P* = 0.0243, one-way ANOVA; note the lower band represents actin) providing strong evidence for the NO-mediated 3-NT/TPI/DHAP/RAGE signaling cascade in prion disease which signifies a disease-associated neurotoxic pathway.

### Nitric Oxide and Glycation Signaling Impact on Prion Misfolding.

**Fig. 5.**
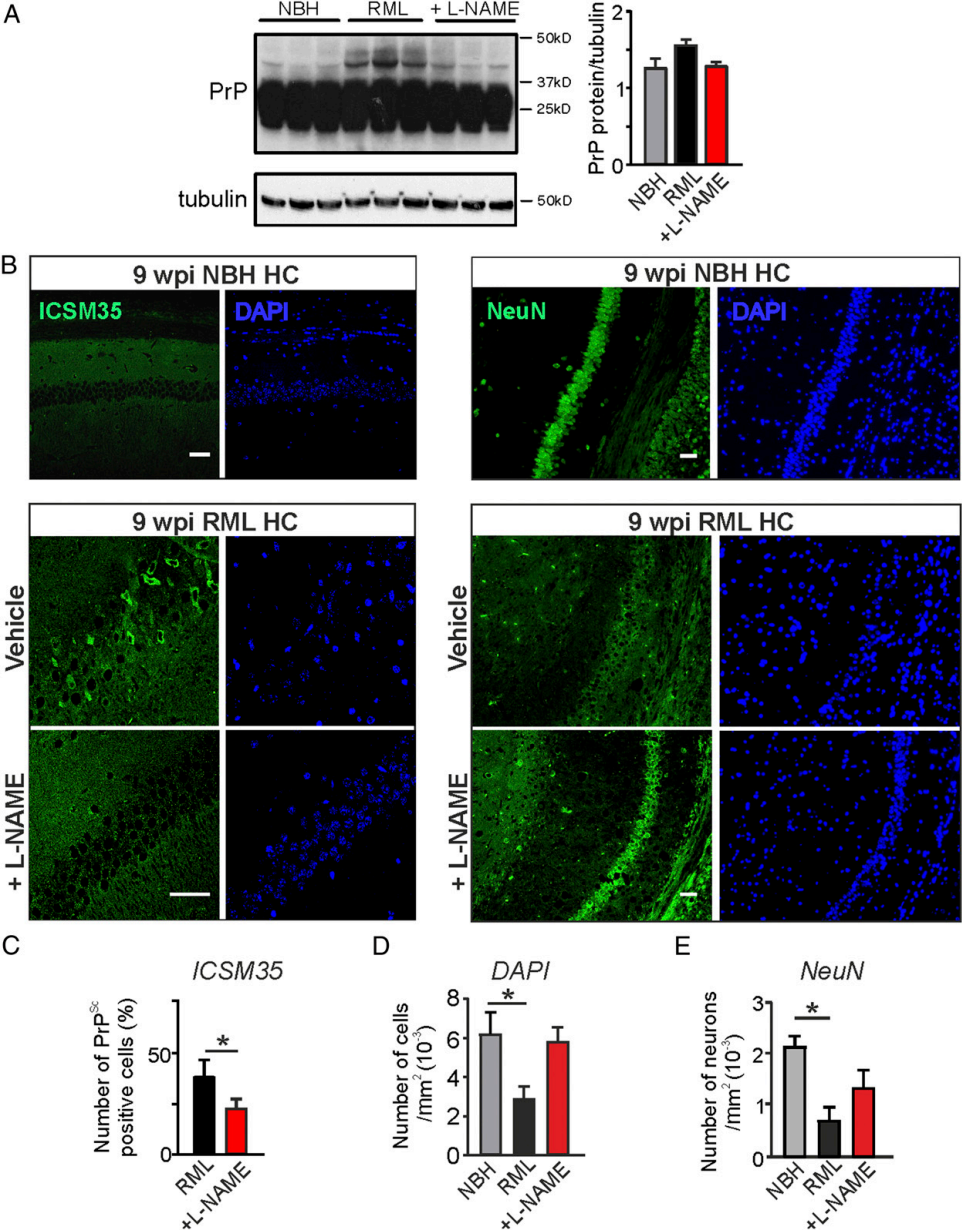
L-NAME treatment mitigates prion protein misfolding and reduces neuronal loss in RML mice. (*A*) Immunoblotting for prion protein in the hippocampus from control mice (NBH), prion-diseased mice (RML), and RML + L-NAME–treated mice at 9 w.p.i. (*B*) (*Left*) Immunocytochemistry of hippocampal regions from NBH, RML, and RML + L-NAME–treated mice (ICSM35+DAPI). (*Right*) Immunocytochemistry of hippocampal regions from NBH, RML, and RML + L-NAME–treated mice (NeuN + DAPI). (Scale bar: 50 μm.) (*C*) Summary of relative numbers of cells positive for PrP^Sc^ signals (number of PrP-positive cells/number of total cells) in the hippocampal CA1 region (Student’s *t* test). (*D*) Total number of cells (DAPI) counted in hippocampal CA1 region under indicated conditions. (*E*) Total number of neurons (NeuN) counted in hippocampal CA1 region under indicated conditions. Data are presented as mean ± SEM, *n* = 3 NBH, *n* = 3 to 5 RML and *n* = 3 RML + L-NAME–treated mice, one-way ANOVA (*A*, *D*, and *E*), *P < 0.05.

The pathological scrapie form of the prion protein is heavily glycated resulting in mainly N^ε^-(carboxymethyl)lysines at up to eight lysine residues, whereas PrP^C^ does not contain glycated amino acids (48, 49). It has been shown that monoglycation or diglycation affects the molecular weight of the prion protein resulting in multiple bands following immunoblotting. We thus tested for expression of prion protein in hippocampal tissues. Interestingly, we noticed the occurrence of bands above the expected PrP^c^ molecular weight (∼36 kDa) in hippocampal samples of RML mice at 9 w.p.i. detected using the ICSM35 antibody raised to specifically recognize β-sheet–rich structures within prion proteins (50) (Fig. 5*A*). These higher-weight bands might therefore reflect glycated and β-sheet–enriched prion protein.

We next assessed hippocampal tissue by immunocytochemistry and stained for prion protein to investigate the localization of the misfolded protein in NBH and RML mice with and without L-NAME treatment. In order to specifically visualize β-sheet–rich PrP^Sc^ that is prerequisite for prion aggregation we immunostained samples using the ICSM35 antibody (50). We detected ICSM35 staining almost exclusively in the cytosol of subiculum neurons in RML mice reminiscent of possible protein aggregates formed within the cytosol (49) (Fig. 5 *B* and *C*). Subiculum areas from NBH mice did not show any labeling in neurons (Fig. 5*B*) confirming PrP^Sc^-specific detection in RML mice. Counting of ICSM35-positive (PrP^Sc^) cells in the hippocampus revealed larger proportions of cells in RML mice compared to RML mice receiving L-NAME treatment, suggesting a reduction in the expression of prion protein misfolding (Fig. 5 *B* and *C*; RML, 38.8 ± 4.1% [*n* = 5 mice]; RML + L-NAME, 23.4 ± 2.7% [*n* = 3 mice], *P* = 0.0346, Student’s *t* test; total cell number from both groups, 679). These data therefore indicate that reducing nitrergic stress could slow down the progression of protein misfolding and/or aggregation.

**Fig. 6.**
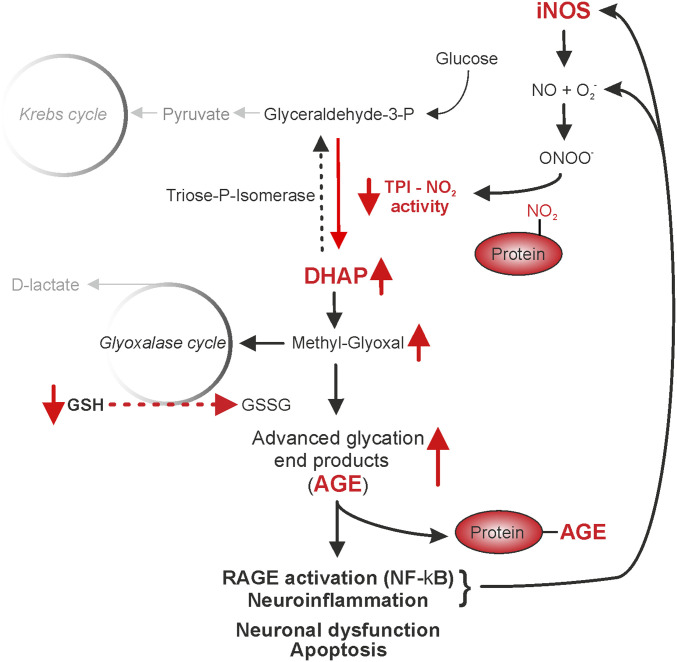
Representation of the pathways impacted on by excessive protein 3-nitrotyrosination. Glycolysis generates glutaraldehyde-3-phosphate (G3P) that the enzyme triose phosphate isomerase (TPI) uses to form dihydroxyacetone (DHAP) which spontaneously and nonenzymatically decomposes to methylglyoxal. The glyoxalase cycle is the detoxification system by which methylglyoxal is transformed into lactate using glutathione (GSH) as a cofactor. Following activation of iNOS associated with enhanced levels of NO and peroxynitrite, 3-nitrotyrosination of TPI decreases its activity, which leads to an accumulation of DHAP and methylglyoxal. High concentrations of methylglyoxal will overload the clearance mechanism, leading to an accumulation of GSSG and the excessive formation of advanced glycation end-products (AGE) with subsequent activation of the receptor for AGE (RAGE). Under pathological conditions, glycation compromises protein functionality and predisposes proteins to aggregation which further contributes to inflammation and cell dysfunction.

Quantification of total cell numbers (DAPI-positive nuclei) within the CA1 region revealed a significant reduction in RML hippocampi at 9 w.p.i. compared to NBH and L-NAME–treated RML mice (Fig. 5 *B* and *D*; DAPI count per μm^2^, NBH, 0.0064 ± 0.001 [*n* = 3 mice]; RML, 0.0029 ± 0.0005 [*n* = 5 mice], *P* = 0.0397; RML + L-NAME, 0.0059 ± 0.0007 [*n* = 3 mice], one-way ANOVA; representative images of DAPI-labeled tissues).

We further confirmed that the absolute numbers of neurons in the CA1 region of hippocampus of L-NAME–treated mice was similar to control NBH hippocampi, whereas vehicle-treated RML mice showed a significant reduction in neuron numbers (Fig. 5 *B* and *E*; NeuN-positive cells per μm^2^, NBH, 0.0021 ± 0.0001 [*n* = 3 mice]; RML, 0.0007 ± 0.0001 [*n* = 5 mice], *P* = 0.0324; RML + L-NAME, 0.0013 ± 0.0003 [*n* = 3 mice], one-way ANOVA; representative images of NeuN-labeled tissues). These data suggest that L-NAME treatment results in a reduction of neuronal loss consistent with the L-NAME–mediated prevention of the decline in neuronal function at this time point of disease (Figs. 2 and 3).

Previously, we reported that the metabolome of the hippocampus as well as the cortex is altered in prion disease at 10 w.p.i. (8). Furthermore, diseased mice exhibit behavioral deficiencies in burrowing activity at 9 w.p.i. (36), a behavior associated with hippocampal but also cortical dysfunction (51). In parallel to the hippocampal increases in DHAP levels, we also confirmed enhanced levels of DHAP in the cortex of RML mice (scaled intensity, NBH, 0.81 ± 0.07, *n* = 8; RML, 1.07 ± 0.07, *n* = 8; *P* = 0.023, Student’s *t* test; *SI Appendix*, Fig. S4*B*) suggesting a similar up-regulated glycation signaling. To test if we can detect potential signs of prion misfolding in the cortex as well and beneficial effects of L-NAME treatment in cortical regions at 9 w.p.i., we assessed cortical tissues for misfolded prion protein by immunocytochemistry (ICSM35). We found that in the cortex from RML mice, prion protein-positive signals exhibit clusters within the cytosol showing typical punctate localizations as seen in the immunocytochemistry data in the hippocampus reflecting a misfolding protein which was absent in tissue from NBH mice (*SI Appendix*, Fig. S4*B*). The number of cells exhibiting such PrP^Sc^ clusters in RML samples was significantly lower following L-NAME treatment suggesting fewer β-sheet–rich prion protein levels (RML, 95.0 ± 2.2% [*n* = 5 mice]; RML + L-NAME, 24.2 ± 5.1% [*n* = 3 mice], *P* < 0.0001, Student’s *t* test; total cell number, 1,892; *SI Appendix*, Fig. S4*C*). Importantly, assessing the total number of cortical neurons showed that L-NAME treatment prevented the reduction in cell loss in disease (cells per μm^2^, NBH, 0.0022 ± 0.0002 [*n* = 3 mice]; RML, 0.0014 ± 0.00006 [*n* = 5 mice], *P* = 0.0119 vs. NBH; RML + L-NAME, 0.0018 ± 0.00004 [*n* = 3 mice], one-way ANOVA; *SI Appendix*, Fig. S4*D*).

Together, our data provide evidence that in vivo NOS inhibition for 3 wk can reduce the amount of prion protein misfolding which positively impacts on neuronal health. We propose that nitrergic stress exerts its effects via posttranslational 3-nitrotyrosination and associated inhibition of TPI activity leading to an accumulation of glycated proteins. The resulting generation of advanced glycation end-products may impact on prion protein misfolding as AGE-assisted conversion of PrP^C^ into PrP^Sc^ also reported for α-synuclein (25, 26) or the Aβ protein (30).

## Discussion

This study was set out to investigate the role of nitrergic stress in the development of protein misfolding neuropathology using prion-mediated neurodegeneration as a well-established disease model system. We show that prion disease is characterized by a range of dysfunctional pathways involving oxidative stress and neuroinflammatory nitrergic signaling as well as activation of the glycation-AGE signaling cascade. Importantly, we also show that suppressing cytotoxic NO levels throughout early disease stages can diminish the progression of the neuronal dysfunction. This was revealed by a sustained synaptic and neuronal function, prevention of excess glycation signaling, and the reduced generation of misfolded prion protein in the hippocampus and cortex of infected animals following NOS inhibition. Although NO signaling plays an important role in physiological neuronal function including regulation of ion channel activities (39, 52, 53) or aspects of neurotransmission (54, 55), NO dysregulation and nitrergic stress have long been associated with numerous neurodegenerative diseases (3, 9, 56). Its main disease-relevant neurotoxic signaling involves excessive posttranslational protein modifications, such as S-nitrosylation and 3-nitrotyrosination. For instance, it has been shown in various systems that S-nitrosylation of synaptic proteins such as AMPA receptors (57), syntaxin (58), complexin (59), stargazin (60), or dynamin (61) can modify their functions (for review, see ref. 4). Several studies have confirmed that suppressing excess NO production might be beneficial under various conditions of neuroinflammation which is a major hallmark of protein misfolding diseases. As such, targeting NO signaling presents an attractive approach for treatment in neuropathology (9). For instance, a potent iNOS inhibitor, GW274150, has been used in phase II clinical trials to treat migraine, and NOS inhibitors are known to provide neuroprotection in PD (13) or under conditions of cerebral ischemia and cerebral artery occlusions (62, 63). In various models of neurodegeneration, pharmacological inhibition of iNOS produced beneficial effects (14, 15) confirming that reducing nitrergic neurotoxicity can protect the central nervous system (64). In addition, two NOS blockers, L-NNA and L-NAME, have been used in human trials to treat septic shock showing mixed outcomes and thus require further investigations (for review, see ref. 65).

However, the exact downstream targets of NO and its signaling routes in disease remain elusive despite the identification of numerous proteins targets. There is growing evidence that high nitrergic activity in conjunction with enhanced oxidative stress can directly result in activation of glycation signaling (20, 66, 67), presenting a positive feedback loop to promote further cellular stress. Glycation has been detected in AD and CJD in earlier studies (34, 68); however, disease-related 3-NT signaling has only recently been proposed to involve glycation signaling in AD (21). This specific pathway requires the dysregulation of glycolysis via the 3-NT–mediated suppression of TPI activity which results in increased cellular levels of DHAP. DHAP spontaneously decomposes to methylglyoxal, which is a highly reactive α-oxo-aldehyde that can modify both proteins and DNA to form AGEs. Methylglyoxal can be metabolized by the glyoxalase pathway, which requires the presence of GSH (summary pathway, Fig. 6). As our data show reductions in neuronal GSH levels and associated enhanced oxidative stress levels in prion disease (8), we suggest that this detoxifying pathway might be compromised. The downstream consequences of increased AGE levels are not fully understood, but emerging evidence implies that this irreversible modification renders many proteins inactive and susceptible to misfolding such as in the case of α-synuclein (25, 26) or Aβ (30) and further increases oxidative stress due to super oxide dismutase inhibition (69). In our study, we could show that pharmacological suppression of NO signaling, predominantly targeting neuroinflammatory iNOS generated NO, leads to diminished 3-NT of TPI and subsequent reduction of AGE formation, as confirmed by reduced RAGE expression. These preventative effects are associated with diminished levels of misfolded prion protein (PrP^Sc^) and improved neuronal health as measured electrophysiologically and consistent with higher numbers of pyramidal neurons in the hippocampus and cortex of L-NAME–treated animals. Since one mechanism that leads to the stabilization and deposition of PrP^Sc^ fibrils may be the result of specific AGE modifications of PrP^Sc^, it is possible that inhibition of NO-mediated AGE formation might be an approach of slowing PrP^Sc^ misfolding, thereby alleviating the progression of prion disease. This could present an approach to diminish the detrimental neuroinflammatory effects seen in neurodegeneration since our evidence for a prolonged neuronal health as a result of treatment implicates this signaling route as an important contributor to neuronal degeneration (Fig. 6) and highlights nitrergic stress and glycation signaling as putative targets for disease-modifying treatments. To take this study forward it will be important to further dissect the identities of AGEs and downstream RAGE signaling, with particular interest in the positive feedback loop of AGE formation of misfolding proteins and the proneuroinflammatory contributions of RAGE signaling.

## Supplementary Material

Supplementary File

## Data Availability

All study data are included in the article and *SI Appendix*.

## Change History

March 16, 2021: The license for this article has been updated.
