# Supplementary material for: Inhibition of neuroinflammatory nitric oxide signaling suppresses glycation and prevents neuronal dysfunction in mouse prion disease
Source: Proc Natl Acad Sci U S A. 2021 Mar 2;118(10):e2009579118. doi: 10.1073/pnas.2009579118 (PMC7958397; doi:10.1073/pnas.2009579118)
Supplement: Supplementary File [file pnas.2009579118.sapp.pdf]

SI Appendix

Inhibition of neuroinflammatory nitric oxide signalling suppresses glycation and prevents neuronal dysfunction in mouse prion disease

Julie-Myrtille Bourgognon[a], Jereme G. Spiers[b], Sue W. Robinson, Hannah Scheiblich[c], Paul Glynn, Catharine Ortori[d], Sophie J. Bradley[e], Andrew B. Tobin[e], Joern R. Steinert[f,1]

Methods

Liquid chromatography–mass spectrometry (LC/MS) analysis

L-NAME was injected (20 mg/kg; *i.p.*) daily for 5 days to reach steady-state levels before the mice were sacrificed and the brains dissected and snap-frozen in liquid nitrogen. The tissue samples were weighed still in the frozen state and disrupted using a micropestle in an Eppendorf tube. A two-stage extraction was performed to ensure denaturation, membrane disruption and extraction of the metabolite L-NOARG. A mix of chloroform/methanol (0.5 ml, 2:1) was added to the tubes and vortexed for 20 min. After centrifugation, the supernatant was removed to another Eppendorf tube and the pellets were re-suspended in 0.5 ml 50% methanol/water. They were again vortexed and re-centrifuged. The solvent mixes were pooled for each sample and then dried in a centrifugal evaporator. Samples were reconstituted in 100% methanol by vortexing and re-centrifugation. The HPLC system used was a modular LC Shimadzu LC system, equipped with pump, cooled autosampler and column oven at 45°C. A Phenomenex Gemini 18 3µ column 100x2mm was used at 45°C, with mobile phase A (0.01% formic acid (pH 4.6) in water) and mobile phase B acetonitrile. The compound was eluted on a gradient from 0.0% B to 60% B in 5 min with a 2 min wash at 100% B. The MS system used was a Sciex 4000 QTrap run in ES+ve MRM mode. Four MRM channels (220.143>87.000, 220.143>174.200, 220.143>59.100 and 220.143>70.200) were monitored to ensure specificity. Quantification was performed against a dilution series of the standard and the extraction efficiency was included in the calculations.

Western blotting

Primary antibody details were as following: complexin1/2, 1:2000, 122102 Synaptic Systems; Kv3.1, 1:2000, Alomone labs; synaptobrevin, 1:1000, ab77314 Abcam; synaptophysin, 1:1000, 5467, Cell Signaling; synapsin, 1:2000, ab64581, Abcam; SNAP-25, 1:1000, ab41726 Abcam; Munc 18, 1:1000, ab3451, Abcam; 3-NT, 1:5000, ab61342, Abcam; TPI, 1:1000, ab6671, Abcam; RAGE, 1:1000, ab3611, Abcam; ICSM35 1:1000, D-Gen Ltd; tubulin 1:5000, 2144, Cell Signaling; actin, 1:5000, ab8224, Abcam.

Immunocytochemistry

Sections were incubated with an anti-PrP antibody (1:1000, ICSM35, D-Gen) to recognise preferentially PrP^{Sc} over PrP^{C}, an anti-GFAP (1:1000, ab134436, Abcam) or an anti-NeuN (1:1000,

MAB377, Millipore). Sections were washed three times and incubated with Alexa Fluor fluorescent secondary antibody for 1 hour at RT in blocking buffer. Following three washes, slices were mounted in Vectorshield hardset mounting medium with/without DAPI (Santa Cruz). For cell number analysis, pyramidal neurons were counted in hippocampal or cortical sections, normalised to area and data averaged from three brains per condition.

qRT-PCR

The target primer/probes used were FAM-labelled *Ncf1* (Mm00447921_m1), *Nfkbia* (Mm00477798_m1), *Nos2* (Mm00440502_m1), *Ppargc1a* (Mm01208835_m1), and *Stip1* (Mm00489584_m1). Reactions were multiplexed and normalised to a VIC-labelled primer/probe for β-actin (Actb; Mm00607939_s1). Relative mRNA expression in each region was determined by normalising to the average amount of hippocampus or cortex from NBH mice using the ΔΔCT method.

Electrophysiology

All electrophysiology studies were performed as reported previously (1): patch pipettes were pulled from glass capillaries (GC150F-7.5, o.d. 1.5 mm, Harvard Apparatus, Edenbridge, UK) and had resistances of 3.5–5 MΩ when filled with the pipette solution. Series resistances were between 12 and 18 MΩ (series resistance compensation and prediction were set at 70%). Data were recorded using a Multiclamp 700B amplifier (Molecular Devices, Sunnyvale, CA, USA). Stimulation, data acquisition, and analysis were performed using pClamp 10.4 and Clampfit 10.4 (Molecular Devices). An artificial cerebrospinal fluid (aCSF) was used for slice incubation, maintenance after slicing, and perfusion during recordings (125 mM NaCl, 2.5 mM KCl, 26 mM $NaHCO_3$, 10 mM glucose, 1.25 mM NaH_2PO_4, 2 mM sodium pyruvate, 3 mM myo-inositol, 2 mM $CaCl_2$, 1 mM $MgCl_2$, 0.5 mM ascorbic acid, pH 7.4 when gassed with 95% O_2, 5% CO_2). Osmolarity was adjusted to 310 mosmol/l. A low-sodium aCSF was used during preparation of slices, with a composition as above for aCSF, except that NaCl was replaced by 200 mM sucrose, and $CaCl_2$ and $MgCl_2$ were changed to 0.1 mM and 4 mM, respectively. The pipette solution for whole-cell recordings contained 120 mM potassium methanesulfonate or caesium chloride, 10 mM HEPES, 0.2 mM EGTA, 4 mM K-ATP, 0.3 mM Na-GTP, 8 mM NaCl, 10 mM KCl, pH 7.4 with osmolarity between to 280-290 mosmol/l. Synaptic stimulation at the Schaffer collateral was achieved using an isolated stimulator (Digitimer, Welwyn Garden City, UK; 1–10 V, 0.1–0.2 ms) *via* a bipolar platinum electrode.

Potassium and sodium currents were recorded in CA1 pyramidal neurons by whole-cell voltage clamp, action potentials (AP) in current clamp. Holding potentials were -60 mV and voltage command steps ranged from -110 mV to +50 mV (200 ms) for potassium current measurements and pre-pulse potentials (400 ms) ranged from -110 mV to +30 mV for sodium current recordings. APs were evoked by 50 pA-step current injections ranging from -100 pA to +400 pA. Cumulative postsynaptic current analysis: The apparent size of the ready releasable pool of vesicles (RRP) was probed by the method of cumulative evoked excitatory postsynaptic current

(eEPSC) amplitudes. Stimulus trains (30 Hz for 1 s) were calculated as the difference between peak and baseline before stimulus onset of a given eEPSC. Receptor desensitization was not blocked as it did not affect eEPSC amplitudes, because a comparison of the decay of the first and the last eEPSC within a train did not reveal any significant difference in decay kinetics. The number of release-ready vesicle pool size was obtained by back extrapolating a line fit to the linear phase of the cumulative eEPSC plot to time zero.

Ultrahigh performance liquid chromatography-tandem mass spectroscopy (UPLC–MS/MS)
Mass spectrometry studies were performed by Metabolon as described previously (1). Further steps were applied: the extract was divided into five fractions: two for analysis by two separate reverse phase (RP)/UPLC-MS/MS methods with positive ion mode electrospray ionization (ESI), one for analysis by RP/UPLC-MS/MS with negative ion mode ESI, one for analysis by HILIC/UPLC-MS/MS with negative ion mode ESI and one sample was reserved for backup. Samples were placed briefly on a TurboVap® (Zymark) to remove the organic solvent. The sample extracts were stored overnight under nitrogen before preparation for analysis. The platform used by Metabolon integrates the chemical analysis, including identification and relative quantification, data reduction, and quality assurance components of the process.

Supplementary Figures

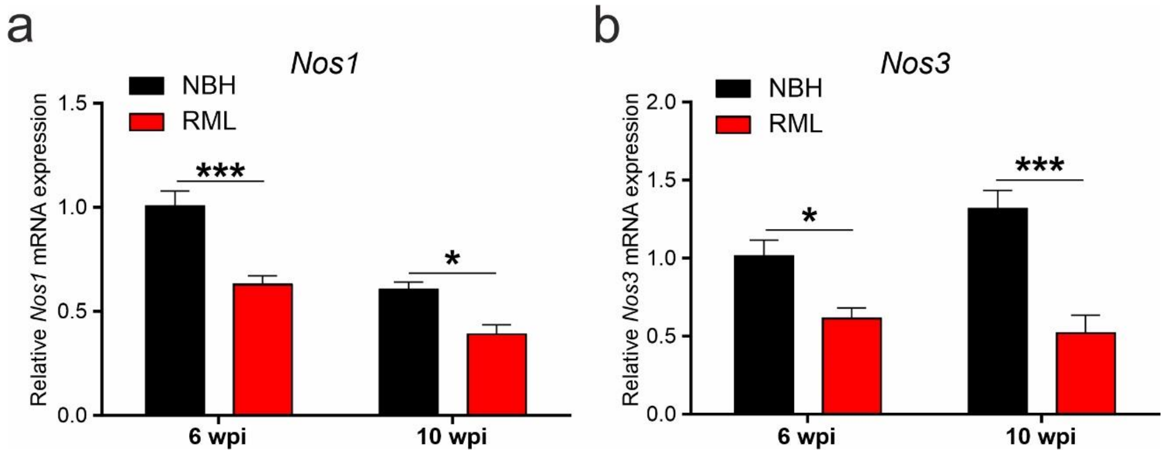

Figure S1. Neuronal and endothelial NOS mRNA levels are reduced in early and late phase prion disease. mRNA levels of the neuronal NOS (a, *Nos1*) and endothelial NOS (b, *Nos3*) were reduced at 6 and 10 w.p.i. (*Nos1*: 6 w.p.i.: NBH: 1.0 ± 0.07, RML: 0.6 ± 0.04, P=0.0003, 10 w.p.i: NBH: 0.06 ± 0.03, RML: 0.4 ± 0.05, P=0.0183; *Nos3*: mRNA levels 6 w.p.i.: NBH: 1.0 ± 0.1, RML: 0.6 ± 0.6, P=0.0281; 10 w.p.i.: NBH: 1.3 ± 0.1, RML: 0.5 ± 0.1, P=0.0004) Data are presented as mean ± SEM, two-way ANOVA, *P<0.05, n=3-4 mice.

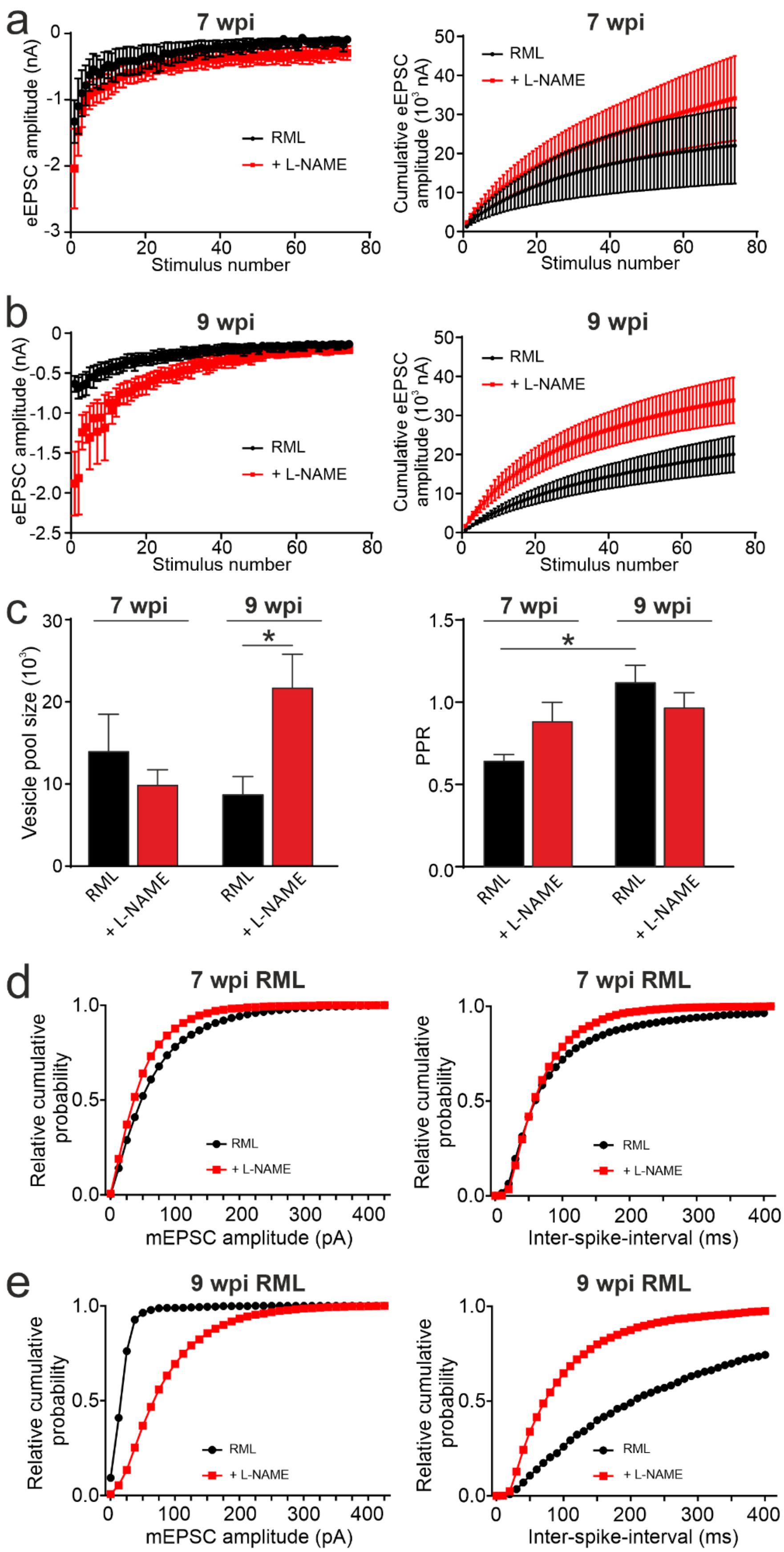

a
7 wpi
eEPSC amplitude (nA)
Stimulus number
RML
+ L-NAME
Cumulative eEPSC amplitude (10³ nA)
b
9 wpi
c
Vesicle pool size (10³)
PPR
*
RML
+ L-NAME
d
7 wpi RML
Relative cumulative probability
mEPSC amplitude (pA)
Inter-spike-interval (ms)
e
9 wpi RML

Figure S2: Decline in hippocampal synaptic strength in RML mice is prevented by NOS antagonism. a, mean eEPSC amplitudes (left) and cumulative amplitudes (right) at 7 w.p.i. for RML mice and RML mice following L-NAME treatment. b, mean eEPSC amplitudes (left) and cumulative amplitudes (right) at 9 w.p.i. for RML mice and RML mice following L-NAME treatment. c, left, estimation of vesicle pool sizes from back-extrapolated eEPSC train amplitudes to time point 0 ms at both ages for RML mice and L-NAME treated RML mice. Right, paired pulse ratios (PPR) at 33 ms stimulus interval for RML and L-NAME treated RML mice. d, relative cumulative probability diagrams shown for mEPSC amplitudes (left) and inter-spike intervals (right) at 7 w.p.i. for RML mice and RML mice following L-NAME treatment. e, relative cumulative probability diagrams shown for mEPSC amplitudes (left) and inter-spike intervals (right) at 9 w.p.i. for RML mice and RML mice following L-NAME treatment. L-NAME treatment results in a significant shift of the data distributions for both parameters at 9 w.p.i. ($P<0.0001$, Kolmogorov-Smirnov test). Data are presented as mean ± SEM, n=4 NBH, n=9 RML and n=6 RML+L-NAME treated mice with n=4-10 neurons per mouse, two-way ANOVA (c), $^*P<0.05$.

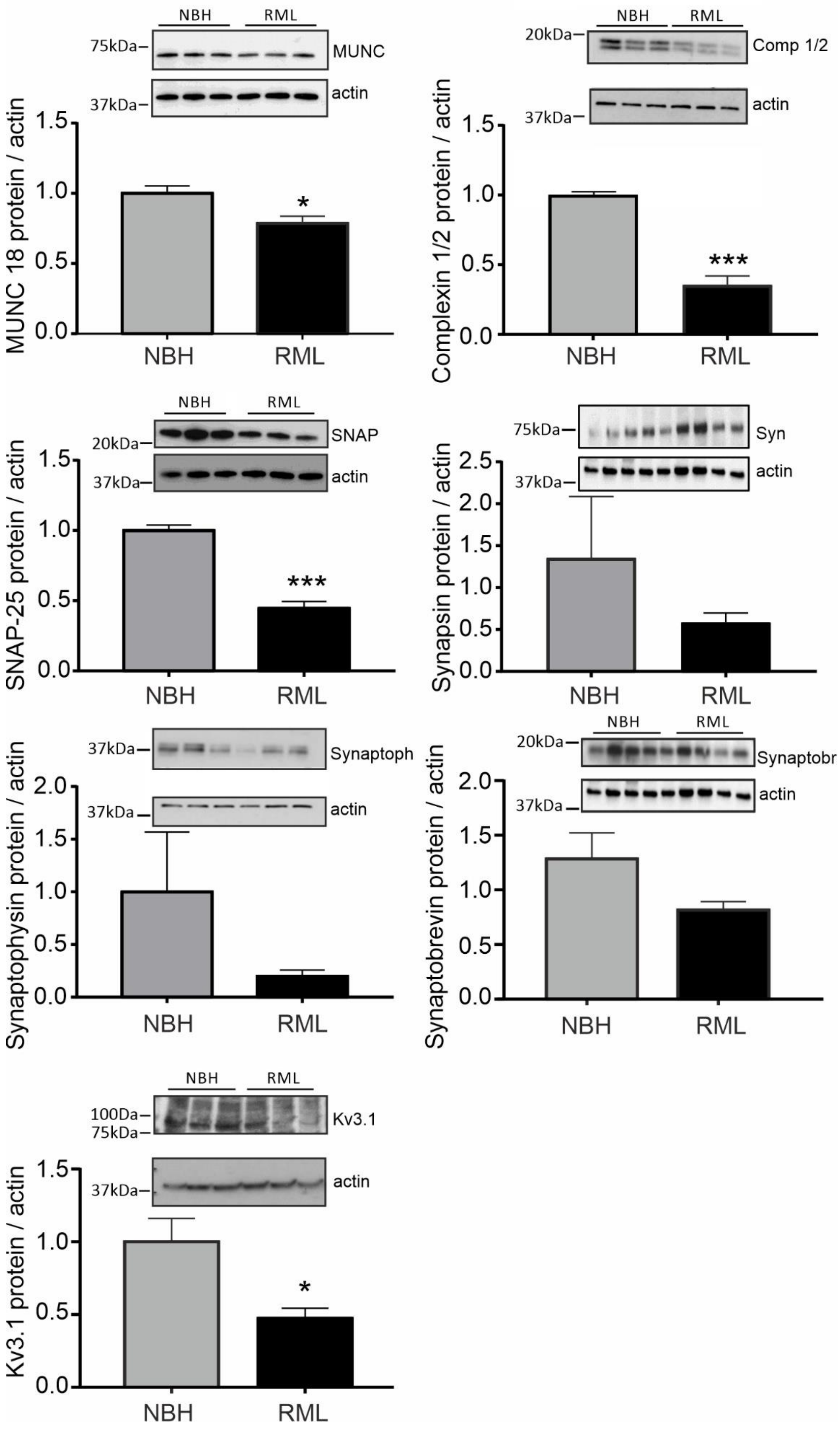
NBH
RML
75kDa
MUNC
37kDa
actin
MUNC 18 protein / actin
1.5
1.0
0.5
0.0
*
NBH
RML
20kDa
Comp 1/2
37kDa
actin
Complexin 1/2 protein / actin
***
20kDa
SNAP
37kDa
actin
SNAP-25 protein / actin
***
75kDa
Syn
37kDa
actin
Synapsin protein / actin
2.5
2.0
1.5
1.0
0.5
0.0
37kDa
Synaptoph
37kDa
actin
Synaptophysin protein / actin
2.0
20kDa
Synaptobr
37kDa
actin
Synaptobrevin protein / actin
100Da
75kDa
Kv3.1
37kDa
actin
Kv3.1 protein / actin
*

Figure S3. Hippocampal neuronal protein expression is reduced in prion disease. Proteins levels of MUNC 18, complexin 1/2, SNAP-25, and Kv3.1 are reduced in prion disease at 9 w.p.i.. Further proteins, including synaptobrevin, synaptophysin and synapsin show tendencies for reduced expression levels in prion disease at 9 w.p.i.. Data are presented as mean ± SEM, n=9 NBH and n=6 RML mice. Some ratio calculations may use the same actin blots. Unpaired Student's test, *P<0.05, ***P<0.001.

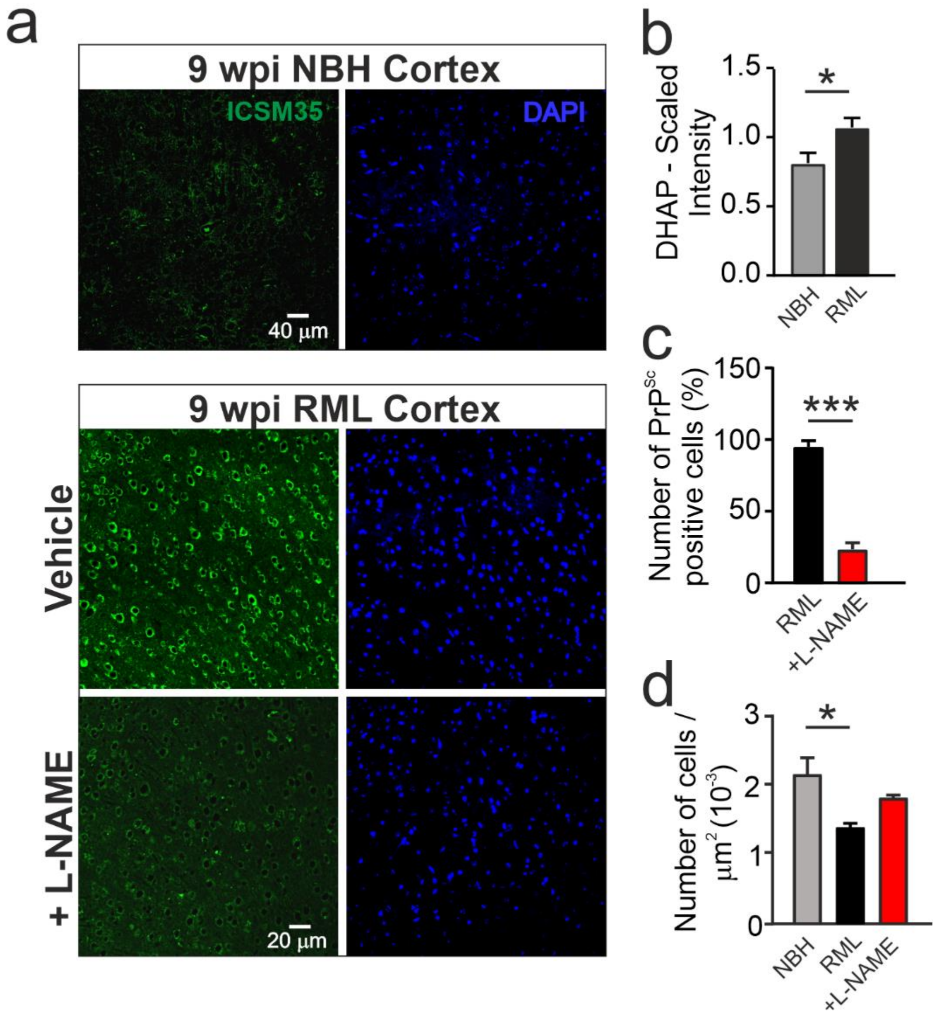

Figure S4. Cortical pathology markers in prion disease are diminished by NOS antagonism. a, immunocytochemistry in cortical regions from NBH, RML and RML+L-NAME treated mice shows staining for ICSM35 and DAPI. b, DHAP levels are increased in the cortex of RML mice. c, summary of relative numbers of cells possessing PrP^{Sc} positive signals (number of PrP positive cells/number of total cells). d, total number of cells counted (DAPI) under indicated conditions. Data are presented as mean ± SEM, n=3 NBH, n=3-5 RML and n=3 RML+L-NAME treated mice. Unpaired Student's *t*-test (b, c), one-way ANOVA (d), *P<0.05, ***P<0.001.

Reference:

1. J. M. Bourgognon *et al.*, Alterations in neuronal metabolism contribute to the pathogenesis of prion disease. *Cell Death Differ* **25**, 1408-1425 (2018).
